# *Acidovorax temperans* skews neutrophil maturation and polarizes Th17 cells to promote lung adenocarcinoma development

**DOI:** 10.1038/s41389-024-00513-6

**Published:** 2024-04-03

**Authors:** Joshua K. Stone, Natalia von Muhlinen, Chenran Zhang, Ana I. Robles, Amy L. Flis, Eleazar Vega-Valle, Akihiko Miyanaga, Masaru Matsumoto, K. Leigh Greathouse, Tomer Cooks, Giorgio Trinchieri, Curtis C. Harris

**Affiliations:** 1 Laboratory of Human Carcinogenesis, Center for Cancer Research, National Cancer Institute, Bethesda, MD 20892 USA; 2 Laboratory Animal Science Program, Laboratory of Human Carcinogenesis, Leidos Biomedical Research, Frederick National Laboratory for Cancer Research, Frederick, MD 21702 USA; 3 Human Science and Design, Robbins College of Health and Human Sciences, Baylor University, Waco, TX 76798 USA; 4 The Shraga Segal Department of Microbiology, Immunology, and Genetics, Ben-Gurion University of the Negev, 84105 Beer-Sheva, Israel; 5 Laboratory of Integrative Cancer Immunology, Center for Cancer Research, National Cancer Institute, Bethesda, MD 20892 USA

**Keywords:** Non-small-cell lung cancer, Microbiology, Tumour immunology

## Abstract

Change within the intratumoral microbiome is a common feature in lung and other cancers and may influence inflammation and immunity in the tumor microenvironment, affecting growth and metastases. We previously characterized the lung cancer microbiome in patients and identified *Acidovorax temperans* as enriched in tumors. Here, we instilled *A. temperans* in an animal model driven by mutant K-ras and Tp53. This revealed *A. temperans* accelerates tumor development and burden through infiltration of proinflammatory cells. Neutrophils exposed to *A. temperans* displayed a mature, pro-tumorigenic phenotype with increased cytokine signaling, with a global shift away from IL-1β signaling. Neutrophil to monocyte and macrophage signaling upregulated MHC II to activate CD4^+^ T cells, polarizing them to an IL-17A^+^ phenotype detectable in CD4^+^ and γδ populations (T17). These T17 cells shared a common gene expression program predictive of poor survival in human LUAD. These data indicate bacterial exposure promotes tumor growth by modulating inflammation.

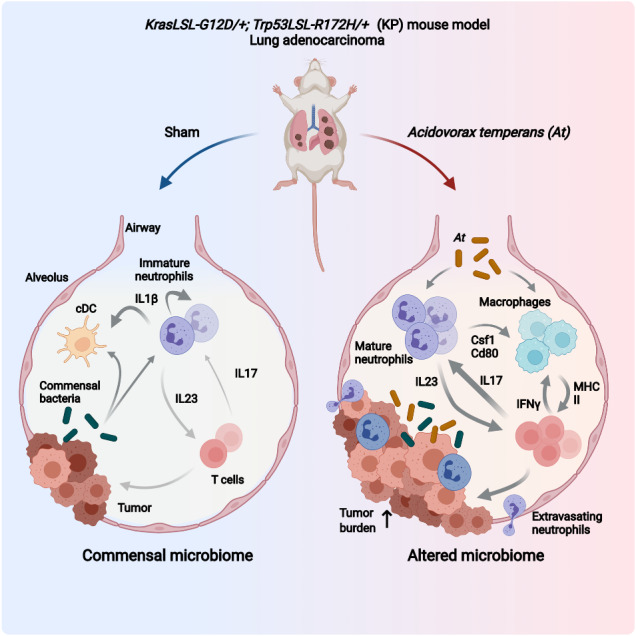

## Introduction

Lung cancer is the leading cause of cancer-specific death in the USA and worldwide [1]. Poor patient outcome is partially due to an inability to predict those patients who are likely to recur [1, 2], thus the identification and development of novel biomarkers is critical. Tobacco smoking is the predominant risk factor for lung cancer, and directly induces tumorigenesis through multiple paths, including carcinogenic metabolites, oxidative stress, and inflammation [3, 4].

Initial immune response to tobacco smoke is driven by IKKβ/NF-κB signaling in macrophages and an increase in proinflammatory cytokines such as IL-1β and IL-6 [5]. Later stage responses see an influx of dendritic cells, neutrophils, and CD4^+^ T-cells [6]. In addition to tobacco smoking, exposure to pathogens is also believed to play a proinflammatory role by creating a local environment primed for oncogenesis. Infection with *Mycobacterium tuberculosis* has been linked to an increased risk for lung cancer development [7–9], possibly through increased infiltration of proinflammatory cells such as neutrophils [10].

Recently, the native microbiota was identified as a key regulator of immune function in an autochthonous mouse model of lung adenocarcinoma (LUAD). Mice kept in specific pathogen-free (SPF) conditions developed more and larger tumors compared to those kept in germ-free (GF) conditions. The presence of bacteria resulted in a proinflammatory microenvironment, characterized by recruitment of IL-1β-secreting alveolar macrophages, which in turn activated IL-17-secreting γδ T cells, finally recruiting large numbers of neutrophils to the tumor, indicating a role for bacteria in tumor growth [11]. In lung cancer patients, lower airway microbes were associated with infiltration of T_H_17 cells and neutrophils [12]. These results suggest an important proinflammatory role for the microbiome in the development of lung cancer.

Our group recently showed that the lung microbiome undergoes dysbiosis in cancer patients [13]. We identified the Gram-negative *Acidovorax* genus as differentially abundant between normal and tumor tissue as well as between lung adenocarcinoma and squamous cell carcinoma. Furthermore, *Acidovorax* abundance was linked to smoking status and *TP53* mutations [13], supported by the detection of *Acidovorax* in tobacco cigarettes [14]. Full-length 16S rRNA gene sequencing as well as fluorescence in situ hybridization identified *Acidovorax temperans* in patient tumors. Our findings were later confirmed by multiple studies, which detected *Acidovorax* 16S signal in both tumor tissue and patient sputum [15–18].

A central question that has emerged from these sequencing-based studies of the microbiome is whether dysbiosis plays a causative or correlative role in tumorigenesis, i.e., is the microbiome a driver or passenger microenvironmental factor? The Jacks lab [11] began to address this question by linking commensal bacteria, inflammation, and LUAD growth. However, the role dysbiosis may play in promoting tumorigenesis is largely unknown. To answer this question, we repeatedly instilled *A. temperans* as a model for dysbiosis into an autochthonous mouse model of LUAD driven by K-ras and Tp53 mutations to mimic the effects of chronic smoking. We found a driver role for *A. temperans* in tumor growth whereby bacterial instillation increased tumor growth through inflammation, primarily driven by neutrophils, macrophages, and CD4^+^ T cells. This proinflammatory response suggests bacterial exposure in the presence of driver mutations in epithelial cells is sufficient to promote a tumorigenic microenvironment.

## Results

### *Acidovorax temperans* exposure accelerates tumor development in an autochthonous LUAD mouse model

**Fig. 1:**
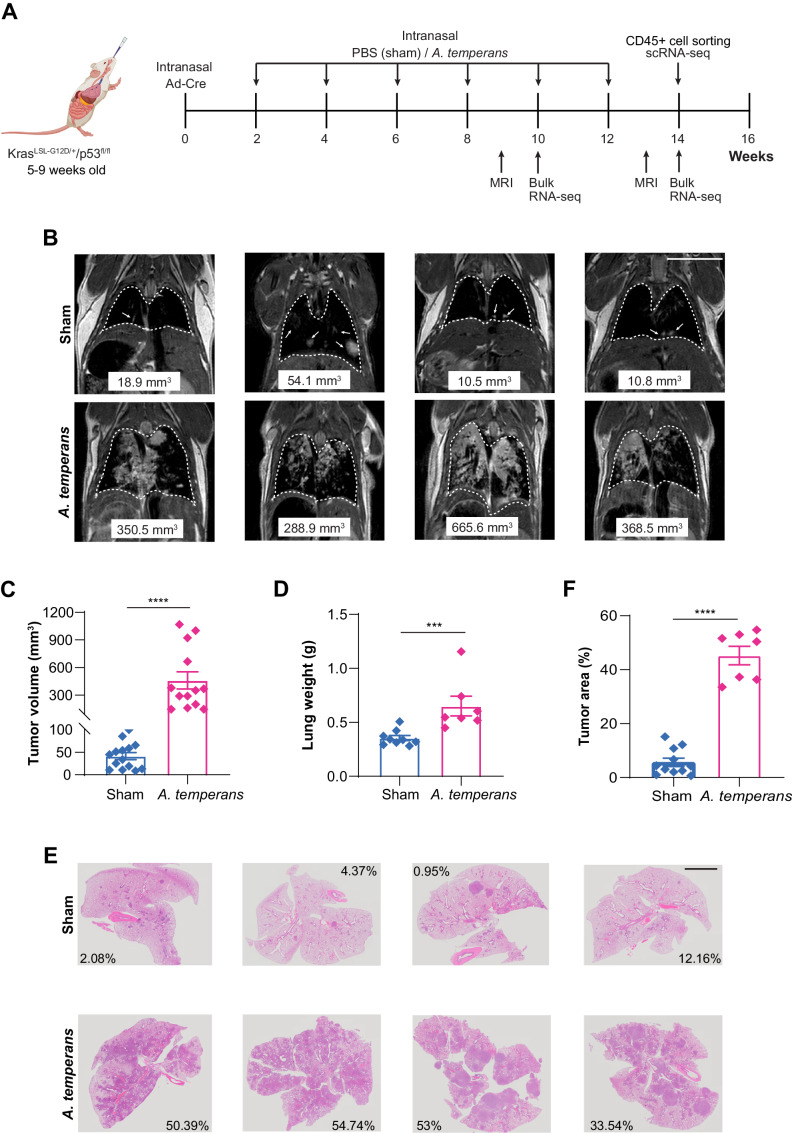
*Acidovorax temperans* accelerates tumor growth in a mouse model of lung adenocarcinoma. **A** Experimental timeline including bacterial dosage schedule. **B** MRI images of sham (1X PBS) (top) and *A. temperans* (bottom) instilled mice at 13 weeks post Adcre. **C** MRI quantification of tumor volume in sham (*n* = 14) and *A. temperans* (*n* = 13) instilled mice. **D** Quantification of lung weight in sham (*n* = 8) and *A. temperans* (*n* = 7) instilled mice. **E** H&E stained images of sham (top) and *A. temperans* (bottom) instilled mice lungs at 13 weeks post Adcre. **F** H&E quantification of tumor area in sham (*n* = 5) and *A. temperans* (*n* = 5) instilled mice. Data presented as mean ± SEM, ***p < 0.001, ****p < 0.0001.

Previous research developed a mutant K-ras and Tp53-driven LUAD mouse model (KP) under the Cre-lox system, resulting in endogenous tumor development reflective of human LUAD [19, 20]. Our group identified *Acidovorax temperans* as enriched in lung cancer which led us to hypothesize that *A. temperans* may play a functional role in lung cancer development [13]. To determine if repeated bacterial exposure, as experienced in chronic tobacco smoking, would result in increased tumor growth, we administrated six biweekly intranasal instillations of PBS (sham) or *A. temperans* in KP mice following Ad-cre instillation (Fig. 1A).

Using non-invasive magnetic resonance imaging (MRI), we measured tumor development at 9- and 13-weeks post instillation (p.i.) of Ad-cre and then sacrificed mice at 14 weeks. We found that at 9 weeks p.i., tumor nodules were only present in *A. temperans* mice (Supplementary Fig. S1A), therefore we focused on the tumor state at 13 weeks p.i. when tumors were visible in both groups. *A. temperans* instilled mice had visibly larger nodules compared to those instilled with sham by MRI and quantification demonstrated an increase in tumor volume (Fig. 1B, C). Consistent with these results, total lung weight was also increased in *A. temperans* mice (Fig. 1D). Tumor area as determined by H&E histology was significantly increased and more high-grade lesions were found in the *A. temperans* mice (Fig. 1E, F, Supplementary Fig. S1B). Taken together, these results revealed that repeated exposure to *A. temperans* could accelerate lung tumor development in the presence of oncogenic K-ras and Tp53 mutations.

We next asked if the accelerated tumor growth we observed could result from *A. temperans* persistence in lungs. We instilled sham or *A. temperans* into mice and homogenized lung tissue at Days 1, 5, and 9 post bacterial instillation for colony plating and enumeration, which revealed a large number of colonies in *A. temperans* mice on Day 1 only (Supplementary Fig. S1C). In contrast, bacterial colony number was comparable between sham mice at each time point and between *A. temperans* mice on Days 5 and 9. We identified a total of seven genera, dominated by *Lactobacillus* and *Streptococcus* spp., while *Acidovorax* colonies were only found in the *A. temperans* mice, and only on Day 1 post instillation (Supplementary Fig. S1D, E). These results indicate *A. temperans* is short-lived in the lungs and is unlikely to colonize this tissue.

### Immune cell infiltration within the tumor microenvironment is altered by *A. temperans*

**Fig. 2:**
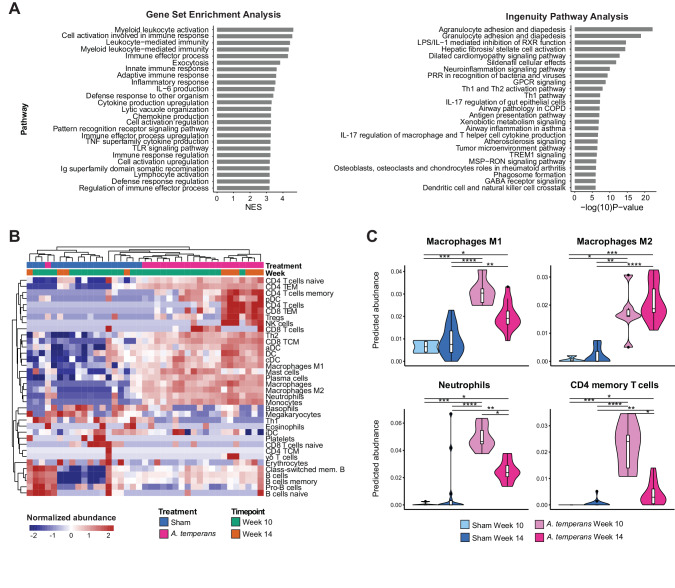
Immune activation differentiates Sham and *A. temperans* instilled mice. **A** GSEA (left) and IPA (right) pathway enrichment in *A. temperans* (*n* = 21) versus sham (*n* = 18) instilled mice across timepoints. **B** Normalized xCell predicted immune cell infiltration in both sham and *A. temperans* instilled mice. **C** Quantification of xCell predicted cell infiltration comparing sham week 10 (*n* = 4), sham week 14 (*n* = 14), *A. temperans* week 10 (*n* = 15), and *A. temperans* week 14 (*n* = 6) instilled mice. Data presented as median value plus quartiles for boxplots, *p < 0.05, **p < 0.01, ***p < 0.001, ****p < 0.0001.

To identify possible mechanisms of accelerated tumor development in *A. temperans* instilled mice, we collected lung tissues from mice at 10 and 14 weeks p.i. and performed bulk RNA-sequencing (Fig. 1A). Pathway enrichment using both GSEA and IPA platforms indicated most pathways upregulated in *A. temperans* mice, regardless of timepoint, were related to immune function (Fig. 2A). We then used xCell to deconvolute the RNA-sequencing data and predict immune cell infiltration in these mice [21]. These results showed that sham and *A. temperans* instilled mice clustered separately, regardless of timepoint (Fig. 2B). Overall, we found proinflammatory cells such as macrophages, dendritic cells, neutrophils, and plasma cells highly enriched in *A. temperans* mice, with effector CD4^+^ T cells and myeloid cells higher at 10 weeks compared to 14 weeks (Fig. 2C). These results suggest that repeated *A. temperans* instillation alters the immune compartment of the tumor microenvironment, dramatically increasing the number of proinflammatory cells.

Previous studies have demonstrated an important role for a proinflammatory tumor microenvironment in KP LUAD development, with dendritic cells [22], macrophages [23], neutrophils [24–27], and T cells [11, 28] all implicated in the etiology of this animal model. Considering the overlap of these cell types with those involved in bacterial response, supported by their enrichment in *A. temperans* mice in our RNA-seq results, we hypothesized that bacterial exposure may accelerate tumor growth by altering the immune microenvironment.

**Fig. 3:**
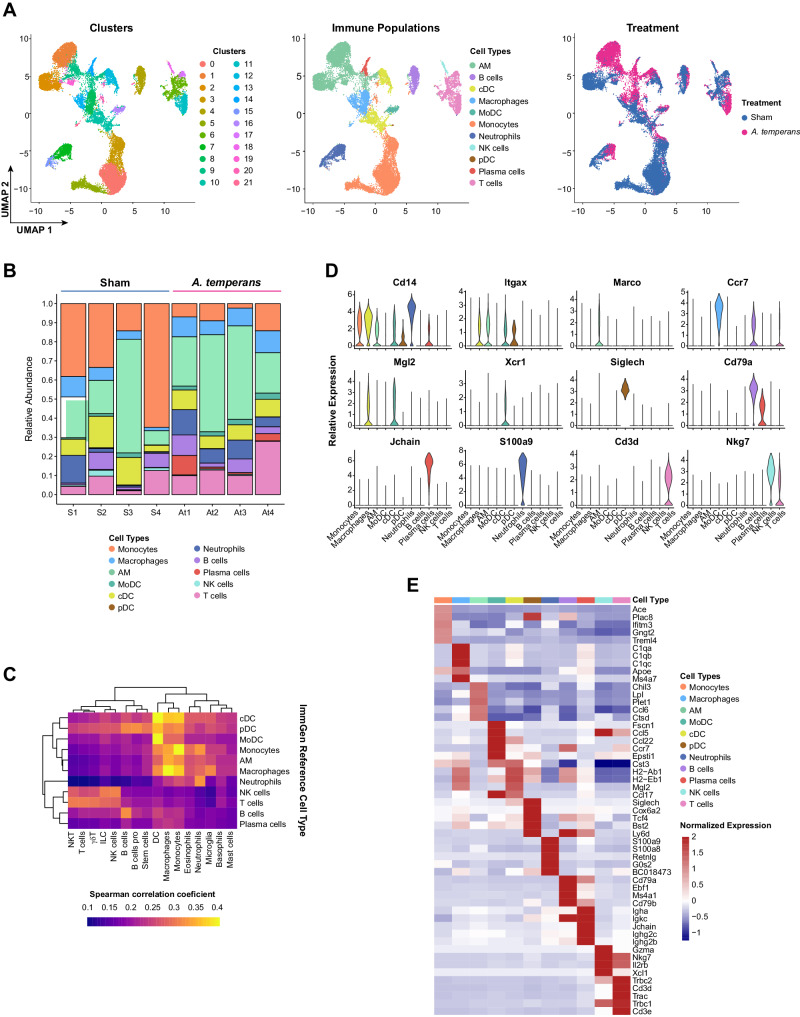
*A. temperans* alters the immune microenvironment in lung adenocarcinoma. **A** UMAP plots of scRNA-seq cell clusters (left), immune cell types (center), and treatment groups (right). **B** Barplot of the relative abundance for each cell type by individual mouse with sham (S, *n* = 4) or *A. temperans* (At, *n* = 4) instillation. **C** Heatmap of ImmGen-based cell type identification. Color scale indicates positive Spearman’s correlation coefficient. **D** Violin plots for marker genes associated with the different cell types. **E** Heatmap of the top five differentially expressed genes for each cell type.

To test this hypothesis, we dissociated lung tissue from four sham and four *A. temperans* instilled KP mice and isolated the CD45^+^ fraction by FACS (Fig. 1A). We then performed droplet-capture single cell RNA-sequencing (scRNA-seq), which returned 25,477 total CD45^+^ cells after filtering. These cells divided into 11 major cell types: monocytes, macrophages, monocyte-derived dendritic cells (MoDC), alveolar macrophages (AMs), conventional dendritic cells (cDC), plasmacytoid dendritic cells (pDC), neutrophils, B cells, plasma cells, NK cells, and T cells (Fig. 3A). Cell types largely overlapped between sham and *A. temperans* mice, although cell proportions showed largest relative shifts from monocyte-high in sham to increased macrophages, AMs, neutrophils, and T cells in *A. temperans* mice (Fig. 3A, B). We identified these clusters through three methods, by comparison to the ImmGen database (Fig. 3C) [29], canonical gene markers (Fig. 3D), and top differentially expressed genes (Fig. 3E, Supplementary Table S1). Collectively, these results demonstrate *A. temperans* alters the immune compartment of the tumor microenvironment in KP mice.

### Lung macrophages upregulate MHC class II in response to *A. temperans*

**Fig. 4:**
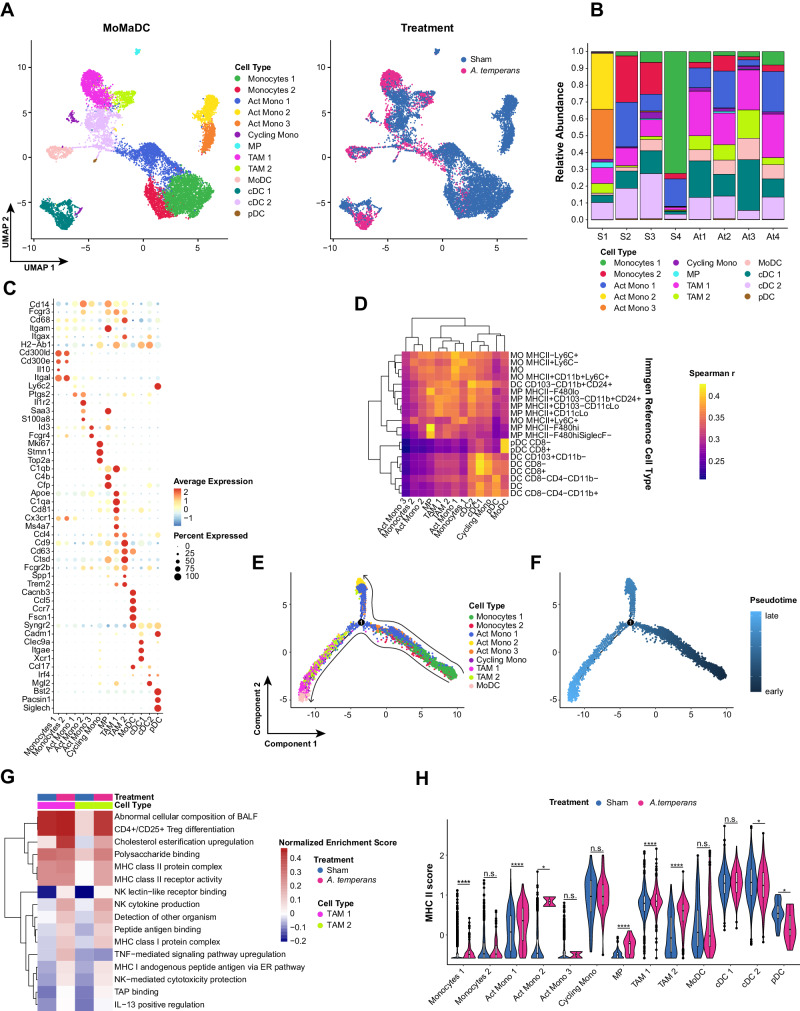
TAMs are expanded and upregulate MHC II in response to *A. temperans*. **A** UMAP plots of monocytes, macrophages, and dendritic cells (MoMaDCs) cell types (left) and treatment groups (right). **B** Barplot of the relative abundance for each cell subtype by individual mouse with sham (S, *n* = 4) or *A. temperans* (At, *n* = 4) instillation. **C** Dotplot of marker genes for each cell type. **D** Heatmap of ImmGen-based cell type identification. Color scale indicates positive Spearman’s correlation coefficient. **E**, **F** UMAP plots of (**E**) trajectory analysis and (**F**) pseudotime projection of monocyte-derived cells. **G** Single-sample GSEA (ssGSEA) heatmap of average normalized enrichment scores for both TAM clusters divided by treatment group. **H** Comparison of average expression of each MHC II component gene (*H2-Aa, -Ab1, -DMa, -DMb1, -DMb2, -Eb1, -Eb2, -Oa, -Ob*) by treatment for each cell type. Data presented as median value plus quartiles for boxplots, n.s. not significant, *p < 0.05, ****p < 0.0001.

Myeloid cells are the first cells to respond to bacterial lung infections and often secrete proinflammatory cytokines linked to tumor development, leading us to first characterize this compartment. We identified 13 subclusters within the scRNA-seq dataset corresponding to monocyte, macrophages, and dendritic cells (MoMaDCs) (Fig. 4A, B). Overall, we identified two clusters of naïve monocytes (*Cd14*+, *Fcgr3-*), three activated monocyte clusters (Act Mono; *Cd14*+, *Fcgr3*+), one cycling monocyte cluster (*Mki67*+*, Stmn1*+*, Top2a*+), three macrophage clusters (*Cd68*+), and four DC clusters (*Syngr2*+) (Fig. 4C, Supplemental Table S3). Within the macrophages, we identified two clusters of tumor-associated macrophages (TAMs; *Fcgr2b*+*, Ccl4*+*, Trem2*+) [30] and one enriched in complement genes (*C4b*+*, Cfp*+*, C1qb*+). Within the DCs, we identified MoDCs (*Ccl5*+*, Ccr7*+*, Fscn1*+), conventional DCs clusters cDC1 (*Clec9a*+*, Itgae*+*, Xcr1*+) [31] and cDC2 (*Mgl2*+*, Irf4*+), and plasmacytoid DCs (*Bst2*+*, Pacsin1*+*, Siglech*+) (Fig. 3C, Supplementary Table S2) [32]. Cell type identification was confirmed by comparison to the ImmGen database (Fig. 4D). We then asked if developmental trajectory followed the conventional route from circulating monocytes to macrophages and DCs. First removing the cDC and pDC clusters as these cell types are not monocyte derived, this analysis revealed that the TAMs and MoDCs were the latest in pseudotime (Fig. 4E, F).

Macrophages have traditionally been classified as M1 or M2, with classically activated M1 macrophages inducing inflammation against pathogens and tumor cells while M2 macrophages are immunosuppressive. Neither TAM cluster showed expression of M1 or M2 macrophage markers (Supplementary Fig. S2A). To better understand differences in their function, we examined changes in TAM gene expression with single-sample gene set enrichment analysis (ssGSEA) [33]. These clusters were differentiated by cholesterol esterification upregulation in TAM-1 in both treatment groups while TNF signaling was specifically upregulated in TAM-1 in response to *A. temperans* (Fig. 4G). Both TAM-1 and TAM-2 clusters were highly enriched for both MHC class I and II antigen presentation in response to *A. temperans* instillation. To determine if MHC upregulation was consistent among monocytes and DCs, we calculated signature scores for all MHC class I and II genes. This revealed *A. temperans* caused upregulation of MHC I in macrophages and DCs, but only macrophages displayed consistent upregulation of MHC II, with TAM-2 cells having the greatest relative increase (Fig. 4H, Supplementary Fig. S2B, C). Similarly, analysis of the alveolar macrophage (AM) compartment revealed low expression of M1/M2 genes while MHC I and II were both broadly upregulated across AMs in response to *A. temperans* (Supplementary Fig. S3, Supplementary Table S3). Together, these results indicate that bacterial exposure induces a broad MHC II response across lung macrophages, potentially contributing to increased tumor growth through CD4^+^ T cell activation.

### *A. temperans* increases tumor-associated neutrophils which express antimicrobial gene programs

Neutrophils are the most abundant immune cell type in human NSCLC and KP mice [26, 34] and have dual function in cancer development and infection response, particularly secretion of proinflammatory cytokines, ROS production, and immunosuppression [35]. These factors implicate neutrophils as essential mediators of early tumor development; therefore, we asked how *A. temperans* altered expression and function of neutrophils in KP mice.

**Fig. 5:**
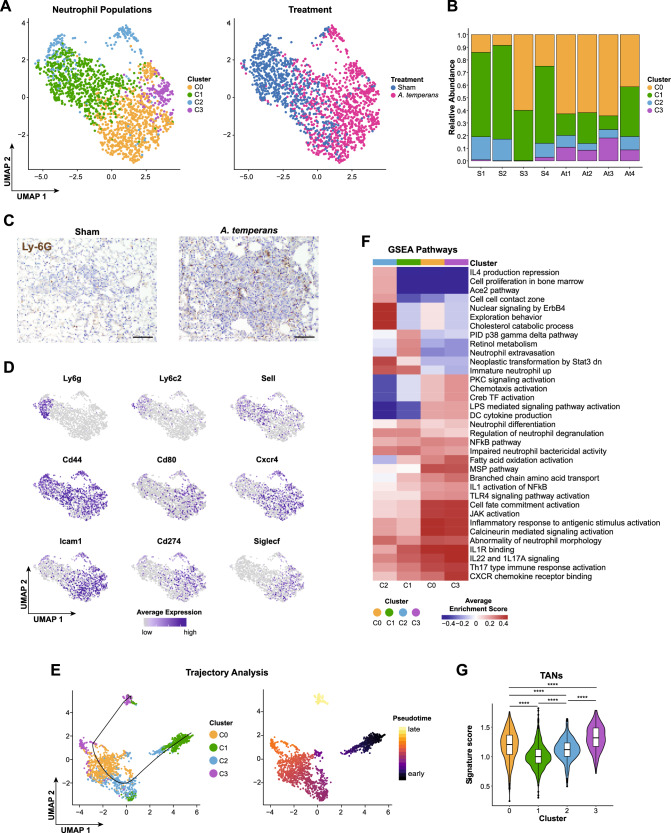
Tumor-associated neutrophils are associated with increased anti-bacterial function. **A** UMAP plots of neutrophil clusters (left) and treatment groups (right). **B** Barplot of the relative abundance for each cluster by individual mouse with sham (S, *n* = 4) or *A. temperans* (At, *n* = 4) instillation. **C** Representative immunohistochemistry images of neutrophil populations (Ly-6G) in sham (left) and *A. temperans* (right) instilled mice. Scale bar 100 µm. **D** Density plots of marker gene expression. **E** UMAP plots of trajectory analysis (top) and pseudotime (bottom) of neutrophil clusters. **F** ssGSEA heatmap of average normalized enrichment scores for each neutrophil cluster. **G** Gene signature scores for tumor associated neutrophils (TANs). TAN signature from accession number GSE118245 [40]. Boxplots indicate median and quartile scores. ****p < 0.0001.

We identified four clusters of neutrophils from the scRNA-seq data, with sham mice having greater proportions of clusters C1 and C2 while *A. temperans* instilled mice greatly increased the cell numbers in clusters C0 and C3 (Fig. 5A, B). We then confirmed an increase in total neutrophils in *A. temperans* instilled mice by immunohistochemistry staining of Ly-6G (Fig. 5C). Markers associated with circulating neutrophils (*Sell*^*hi*^, CD62L; *Cxcr4*^*lo*^) were higher in clusters C1 and C2 while markers linked to increased effector function (*Icam1*), immunosuppression (*Cd274*+, PD-L1), and tumor-promotion (*Siglecf*) were higher in clusters C0 and C3; these expression patterns generally corresponded with treatment group (Fig. 5D, Supplementary Table S4) [36, 37]. To verify this change in marker gene expression from control to bacterial exposure, we performed trajectory analysis on the scRNA-seq clusters. This revealed C1 was earliest and C3 latest in pseudotime (Fig. 5E), consistent with expression of these marker genes.

To further understand transcriptional differences between sham and *A. temperans*-associated neutrophils, we performed ssGSEA, which suggested C2 represented the most immature cell state, with high enrichment scores for cell proliferation in bone marrow, immature neutrophils, and cholesterol catabolism, important for neutrophil development and release from the bone marrow (Fig. 5F) [38]. C1 pathways were enriched for neutrophil extravasation while activated, effector functions such migration, chemotaxis, and bacterial response and killing, were primarily associated with C0 and C3, underlined by an LPS response which activated cytokine production.

We then asked if these changes reflected gene expression profiles of immunosuppressive neutrophils in cancer. Tumor-associated neutrophils (TANs) are required for tumor progression and metastasis [39], and comparison of a TAN gene signature revealed C3 had the highest expression of this signature (Fig. 5G) [40]. Collectively, these results demonstrate that dysbiosis response is a key programming event for tumor-associated neutrophils, suggesting that these neutrophils, while being responsible for clearing bacteria from the lungs, alter the tumor microenvironment.

### *A. temperans* robustly induces T_H_17 polarization

**Fig. 6:**
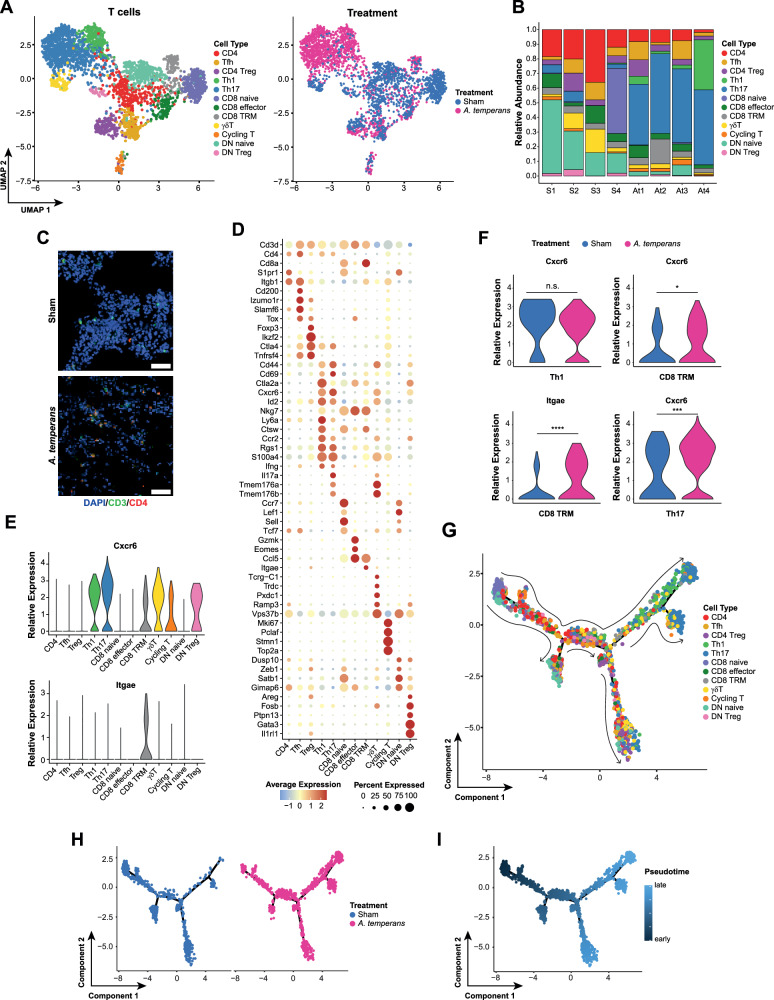
T cells are terminally polarized to a Th17 phenotype. **A** UMAP plots of T cell subtypes (left) and treatment groups (right). **B** Barplot of the relative abundance for each cell subtype by individual mouse with sham (S, *n* = 4) or *A. temperans* (At, *n* = 4) instillation. **C** Representative immunofluorescence images of T cell populations in sham (top) and *A. temperans* (bottom) instilled mice. Blue DAPI, green CD3, red CD4, scale bar 50 µm. **D** Dotplot of marker genes for each cell type. **E** Expression level of tissue residency marker genes by cell type. **F** Expression level of tissue residency markers by cell type and treatment group. UMAP plots of trajectory analysis by (**G**) cell type and (**H**) treatment group. **I** UMAP plots of pseudotime projection of T cells. n.s. not significant, * *p* < 0.05, *** *p* < 0.001, **** *p* < 0.0001.

Having demonstrated large proinflammatory changes in the myeloid compartment driven by MHC upregulation in MoMaDCs, we then examined if these changes were reflected in T cells, as well. We identified a total of 12T cell types and sham mice had higher proportions of naïve T cells while *A. temperans* mice showed greater CD4^+^ effector populations (Fig. 6A, B, Supplementary Table S5), which we confirmed by immunofluorescence (Fig. 6C). These effector populations included follicular helper T cells (Tfh; *Cd200*+*, Izumo1r*+, *Slamf6*+) [41], Tregs (*Foxp3*+*, Ikzf2*+*, Ctla4*+), Th17 (*Il17a*+*, Tmem176a*+*, Tmem176b*+) [42], and Th1 (*Ccr2*+*, Ifng*+). We found three clusters of cells which did not express either *Cd4* or *Cd8a* (Fig. 6D), which corresponded to γδ T cells (*Tcrg-C1*+*, Trdc*+), double negative (DN) naïve (*Ccr7*+, *Lef1*+*, Sell*+, *Tcf7*+), and a DN Treg-like population (*Areg*+*, Gata3*+*, Il1rl1*+) [43].

T cells from *A. temperans* mice also showed greater expression of tissue residency markers *Cxcr6* and *Itgae* (CD103) in CD4^+^ and CD8^+^ T cells (Fig. 6E, F). As tissue residency is associated with effector function, we then asked if these effector CD4^+^ cells represented a terminal cell state. Trajectory analysis which revealed naïve CD8 and DN cells were earliest in pseudotime, while Th1 and Th17 cells were latest (Fig. 6G – I). The similarity of marker genes between the Th1 and Th17 cells, combined with high expression of *Cxcr6* in Th1 and Th17 cells, suggested that effector CD4^+^ T cells acquire a tissue residency phenotype prior to polarization. In support of this hypothesis, *A. temperans* T cells were consistently later in pseudotime than sham (Fig. 6H), suggesting that bacterial exposure is a key factor for establishing CD4^+^ T cell lung residency and subsequent T_H_1/T_H_17 polarization.

### *A. temperans* induces specific IL-17 and broad IFN-γ response in T cells

Previous data examining murine colonic effector T cells suggested that T cell phenotype was shaped by response to specific pathogens [44]. We asked if the T cell polarization induced by *A. temperans* was specific to this species or was consistent with more general microbial dysbiosis. We first performed bulk TCR-seq from lung tissues, which showed a sharp decrease in TCR diversity in *A. temperans* mice (Supplementary Fig. S4A, B), due to bacterial-driven hyperextension of specific clonotypes (Supplementary Fig. S4C–E). We then compared bacterially induced T cell gene signatures from mice infected with either *Citrobacter rodentium* (T_H_17 response) or *Salmonella enterica* serovar Typhimurium (IFN-γ response), both Gram-negative species [44]. Expression of the *C. rodentium* signature was predominantly found in our Th1, Th17, and γδ T clusters (Supplementary Fig. S5A, B). Although the *Salmonella* Typhimurium signature was also highest in Th1 and Th17 clusters, we observed consistently high expression throughout our dataset, but upregulated in *A. temperans* mice compared to sham overall (Supplementary Fig. S5C, D). These data suggest that the Th17 cell cluster we observe is not specific to *A. temperans*; however, the combination of general IFN-γ and specific T_H_17 polarization may represent a specific inflammatory response to this species.

Based on the widespread expression of the *Salmonella* Typhimurium gene signature, we asked if both *Il17a* and *Ifng* were upregulated in response to *A. temperans*. Our results showed *Il17a* and its transcription factor *Rorc* were largely restricted to Th17 and γδ T cells while *Ifng* and its transcription factor *Stat4* were highly expressed in non-naïve T cells (Supplementary Fig. S6A, B). Overall, most T cell subtypes expressed *Ifng*, with nearly half of Th1 and a third of Th17 cells positive for this transcript and expression was elevated in response to *A. temperans* (Supplementary Fig. S6C–E). Within Th17 cells, a subpopulation was double positive for *Il17a* and *Ifng* (Supplementary Fig. S6F, G), a highly inflammatory cell state increased in smokers [45]. These data suggest that *A. temperans* alters the immune microenvironment through multiple signaling pathways which culminate in *Il17a* + /*Ifng* + T cells to greatly increase inflammation.

### A conserved gene signature in T17 cells is predictive of poor survival in LUAD

**Fig. 7:**
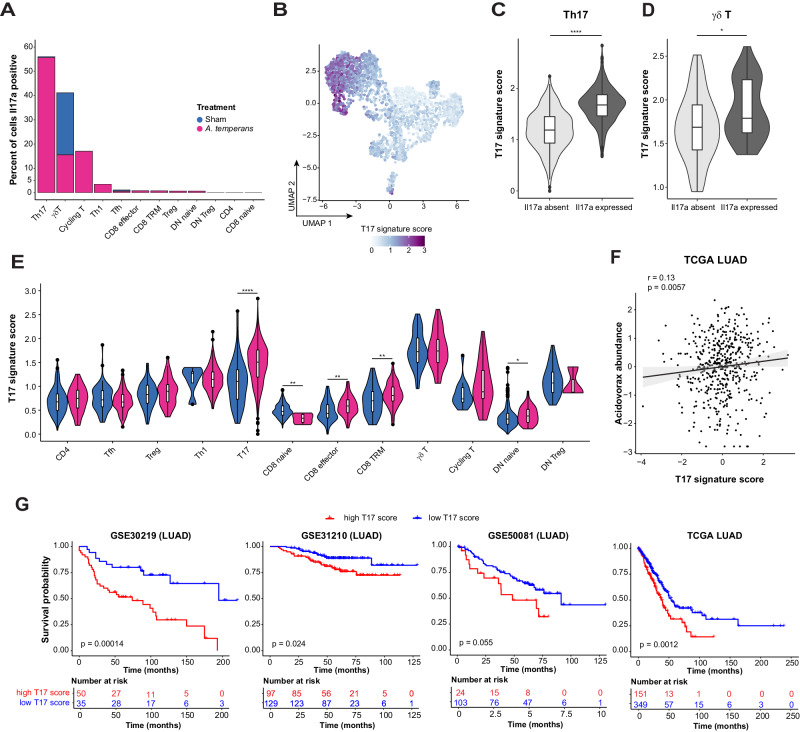
A pan T17 gene signature is predictive of poor prognosis in human LUAD. **A** Percent of cells *Il17a* positive per T cell subtype and treatment group. **B** UMAP projection of a pan T17 cell gene signature within the T cell compartment. Expression of a pan T17 cell gene signature in *Il17a* negative (absent) and positive (expressed) cells within the (**C**) Th17 and (**D**) γδ T clusters. **E** Expression of a pan T17 cell gene signature in all T cell clusters split by treatment group. **F** Correlation of genus-level *Acidovorax* abundance and the pan T17 gene signature score within the TCGA LUAD dataset [13]. **G** Kaplan-Meier curves for survival property within four human LUAD cohorts – GSE30219, GSE31210, GSE50081, and TCGA [74–77]. Boxplots indicate median and quartile scores. * *p* < 0.05, ** *p* < 0.01, **** *p* < 0.0001.

In addition to Th17 cells, approximately 40% of γδ T cells also expressed *Il17a* (Fig. 6D). Examining every T cell cluster revealed that most *Il17a*+ cells, regardless of cell type, were from *A. temperans* mice (Fig. 7A). However, given the high percentage of *Il17a*+ cells in Th17 and γδ T clusters, we hypothesized that gene expression may be similar in both clusters which could then identify a gene set important for IL-17 polarization in pan T cell subtypes (T17). To test this, we combined these two clusters and calculated differentially expressed genes against all other T cells. We then calculated a T17 gene signature score from the expression of each of the genes upregulated in both clusters. The resulting T17 signature was highest in Th17 and γδ T cells, and specifically upregulated in *Il17a*+ cells compared to *Il17a*- cells in both Th17 and γδ T clusters (Fig. 7B, C, Supplementary Table S6). Interestingly, this signature score was higher in *A. temperans* mice in Th17 but not γδ T cells (Fig. 7D). We also observed upregulation in effector CD8^+^ T cells from *A. temperans* mice, which suggests repeated exposure to *A. temperans* induces a T17 polarization in multiple T cell subtypes.

We then asked if this T17 gene signature was important in human lung cancer. Leveraging the metatranscriptomics data that we had previously generated using TCGA LUAD [13], we examined the association of the T17 signature with *Acidovorax* exposure in these patients. The T17 signature score was weakly, but positively, correlated with *Acidovorax* abundance (Fig. 7F), suggesting microbial dysbiosis may also influence T17 polarization in human LUAD. Next, we asked if high expression of the T17 signature was predictive of patient survival. We stratified patients by low or high expression of the T17 signature score in four cohorts of LUAD patients, including TCGA. This stratification revealed high expression of the T17 gene signature was a poor prognostic in LUAD for overall survival (Fig. 7G). These results suggest T17 polarization, regardless of T cell receptor subtype, accelerates tumor development and results in worse survival in patients.

### Cell-cell signaling switches from IL-1β driven to broad proinflammatory activation in response to *A. temperans*

**Fig. 8:**
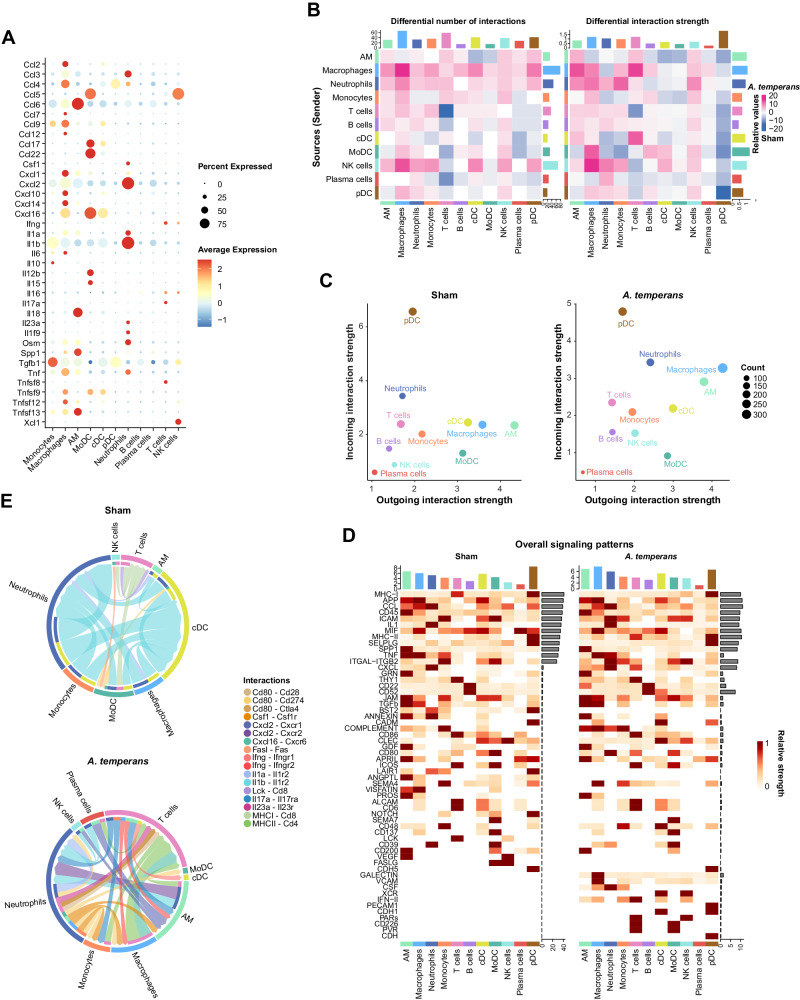
Cell-cell communication demonstrates *A. temperans* induces robust signaling between neutrophils, macrophages, and T cells. **A** Dotplot of cytokine expression by cell type. **B** Heatmap depicting differential interactions as calculated by expression of ligand in outgoing cell type and its cognate receptor in the incoming cell type, with total number (left) and interaction strength (right). Cell-cell interactions enriched in sham mice are indicated by blue and those enriched in *A. temperans* mice indicated by pink. **C** Cross-referenced incoming and outgoing interaction strength for each cell type in sham (left) and *A. temperans* (right) mice. **D** Heatmap of overall signaling patterns by cell signaling pathway for each cell type in sham (left) and *A. temperans* (right) mice. **E** Chord diagrams showing pathways significantly enriched in Sham (top) and *A. temperans* mice (bottom). Chord width indicates aggregate expression of the ligand and receptor, arrow indicates direction from sender to receiver population, outer rings indicate sender cell type, inner rings indicate receiver cell type, and links are colored by interaction pairing. Individual chords are colored by secreting cell type and arrows indicate receptor cell type.

We then investigated cell-cell communication to determine the potential mechanism of *A. temperans*-mediated LUAD progression. Examination of cytokines within all cell types revealed immune cell-of-origin for multiple cytokines previously implicated in KP mouse etiology: IL-1β (neutrophils), IL-23 (neutrophils), and IL-17 (T cells) (Fig. 8A). In contrast to previous results, IL-22 was not detected, and AMs were not a major source of IL-1β or IL-23 [11], suggesting that introduction of an external bacterial species dramatically alters immune cell signaling in KP mice.

We then compared cell-cell communication by examining combined changes in expression of ligand-receptor pairs in sham and *A. temperans* mice. Examination of the total signaling strength (combined incoming and outgoing signal) revealed a specific increase in number of interactions among neutrophils, AMs, macrophages, and T cells in response to *A. temperans* (Fig. 8B, C, Supplementary Tables S7, S8). Sham mice were enriched for BST2, LAIR1, FASLG, IL1B, and VEGF ligand-receptor signaling while *A. temperans* mice were enriched for a MHC class I to class II switch, CXCL, IFN type II, and CSF signaling (Supplementary Fig. S7). In-depth analysis of key ligand-receptor pathways indicated an increase in *Csf1-Csf1r* signaling between neutrophils and monocytes/macrophages; *Ifng/Ifngr1/2* between T cells and monocytes, macrophages, and AMs; *Cxcl2-Cxcr1/2* between neutrophils and AMs; and a strong increase in *MHC II-CD4* between macrophages, cDC, and T cells (Fig. 8D, E). High expression of these cytokines in *A. temperans* instilled mice was validated using our bulk RNA-seq data (Supplementary Fig. S8). *Il17a-Il17ra* interactions were not predicted due to their scRNA-seq expression levels below threshold in sham mice which prevented fold differences from being calculated, demonstrating specific expression in non-γδ T cells in *A. temperans* mice.

Overall, these results indicate that bacterial exposure causes massive inflammation in the KP mouse model of lung cancer, with mature, tumor-associated neutrophils secreting Csf1 to promote differentiation of monocytes to macrophages, which then strongly upregulate MHC class II to stimulate T cells into T17 polarization. This proinflammatory cell infiltration provides mechanistic insights into how dysbiosis alters and promotes lung cancer development, which may have implications for smoking-related tumorigenesis.

## Discussion

Previous studies have demonstrated lung and other cancers feature dysbiotic microbiomes, but a central question is if this dysbiosis contributes to tumor growth [46, 47]. We hypothesized that changes to the microbiome in lung cancer patients likely resulted from repeated exposures through smoking and/or oral microaspirations [48]. To mimic these multiple exposures, we repeatedly instilled *A. temperans*, which we previously identified as associated with smoking and *TP53* mutations in human lung cancer [13], in the KP mouse model of LUAD. This revealed that *A. temperans* increased tumor burden and development, partially through large proinflammatory changes to the tumor microenvironment, driven by tumor-associated neutrophils, MHC class II expressing macrophages, and T17 cells.

Neutrophils and AMs likely form the first-line response to *A. temperans* instillation and secretion of IL-1β and IL-23 from these cells was previously found to be critical for activation of γδ T17 cells in KP mice, which then recruited additional neutrophils to the tumor site [11]. We also found *Il1b* was expressed by monocytes and macrophages but observed that the primary source of *Il1b* was neutrophils themselves, which was secreted in an autocrine loop or to cDCs in sham mice. These differences are likely directly attributable to the instillation of *A. temperans* to the lung, as Jin et al. [11] compared KP mice kept in SPF and GF conditions, identifying γδ T17 cells as the key subpopulation driving inflammation. In keeping both sham and *A. temperans* instilled mice in SPF conditions, our data suggests that rather it is neutrophils that are the primary drivers of inflammation following bacterial exposure. Using an orthotopic mouse model with cancer cells derived from KP mice, Tsay et al. [49] also found a similar pathway in response to the oral commensal *Veillonella parvula*. Following *V. parvula* instillation in the lungs, they reported an increase in T_H_17 and γδ T17 cells which attracted large infiltration of neutrophils. Collectively, these results indicate a key role for bacterially induced T17 cells and neutrophils in the development of murine LUAD.

Mature, SiglecF^high^ neutrophils are characterized by a long intratumoral half-life (3–5 days), lack of proliferation, increased ROS production, and promotion of tumor cell proliferation [25, 36]. The source of these cells is unknown and SiglecF^high^ neutrophils are not found in the bone marrow, circulation, or spleen [36]. Instead, it is thought that these cells complete maturation in the lungs after infiltrating the tumor site. Similarly, immature neutrophils are found in the circulation of inflammation patients and undergo maturation at the site of *Staphylococcus aureus* infections [50, 51]. *Siglecf* expression was increased in response to *A. temperans* instillation and associated with strong immune response gene expression, including LPS response and IL-17 signaling. Our data suggest repeated exposure to even transient bacterial exposure recruits immature neutrophils to the lung where they undergo localized maturation and persistence for tumor promotion, although further studies are needed to test this hypothesis.

Our data have several aspects that are directly relatable to human lung cancer. First, we previously identified *Acidovorax* spp. enriched in smokers and hypothesized that tobacco smoke was a possible means for introducing *Acidovorax* into the lungs [13]. Smoking gradually reduces epithelial barrier function, potentially allowing bacteria such as *Acidovorax* direct access to tumors [52]. *Acidovorax* spp. have been detected in cigarettes and possess genes for catabolism of smoking-associated hydrocarbons [14, 53]. Smoking also results in a similar inflammatory pathway to what we identified in *A. temperans* mice, potentially a response to the large amounts of LPS contained in cigarettes [5, 6, 54, 55]. LPS by itself is capable of accelerating lung cancer growth in vivo, partially through macrophage infiltration and activation of NF-κB and STAT3 signaling [56], providing an additional route for smoking-induced oncogenesis. Second, neutrophils are the most abundant immune cell found in human lung cancer and high infiltration is generally associated with both poor prognosis and resistance to various therapies [34, 50, 57]. Tumor-associated neutrophils, characterized by an activated phenotype of CD62L^low^ (*SELL*)/CD54^high^ (*ICAM1*) (comparable to our scRNA-seq clusters C0 and C3), have high rates of phagocytosis and ROS production, and can directly induce cytokine production in activated T cells [58], which our data suggests could occur through either CD80 or IL-23 expressed by the neutrophils. Third, we identified a gene signature suggestive of pan T17 polarization in response to bacterial exposure in mice that was predictive of poor prognosis in human LUAD. T17 cells contribute to inflammation in NSCLC patients and are a poor prognostic factor [11, 59]. T_H_17 and γδ T17 cells can be isolated from the blood of patients and interestingly tumor resection reduced the number of circulating cells [60], suggesting that an intratumoral source is responsible for inducing polarization. Together, these factors demonstrate that a proinflammatory tumor microenvironment in patients is reflective of the bacterial-associated changes we observe in KP mice.

It is important to note that our data were generated from introduction of a single bacterial species, namely *A. temperans*, to KP mice, which is unlikely to recapitulate the changes observed in human patients and raises questions regarding optimal microbiome study design. Ecological overlap in the context of metabolic operons has been suggested as a hidden driver within the gut microbiome, where individual taxa are dispensable due to functional redundancy, such as short-chain fatty acid production [61, 62]. Dysbiosis in cancer focuses on wholesale changes between non-tumor and tumor tissue and studies rarely, if ever, describe only a handful of changes. However, functional studies of the microbiome typically utilize one of two strategies – addition of a single species (as in this study) or the removal of the microbiome by antibiotics or keeping the animals in GF conditions. A hybrid strategy, as recently described through the creation of an artificial microbiome delivered to GF mice [63], may be the ideal experimental design to study the role(s) of specific taxa in tumor development in a controlled setting.

Our results demonstrate that dysbiosis of the lung microbiome is a contributing factor in tumor development and progression, by promoting large inflammation of the microenvironment, with many known proinflammatory cytokines sharply upregulated in response to *A. temperans*. Further studies are needed with in-depth phenotypic profiling of neutrophil and T17 function in both human and mouse to precisely determine their roles in development of LUAD. Mechanistic understanding of these pathways suggests that anti-neutrophil and IL-17A therapies represent intriguing and promising targets for intervention and development of targeted therapies in LUAD.

## Materials and methods

### Animal model, bacterial culture, and instillation

*Kras*^*LSL-G12D/+*^*; Trp53*^*LSL-R172H/+*^ (KP) mice were purchased from Jackson Laboratory (Bar Harbor, ME) and housed under SPF conditions in accordance with the approved NIH-NCI/CCR animal use protocol (# ASP-19-334) and biosafety protocol (#19-51). For all experiments, male and female KP mice aged 5–9 weeks old were administrated with 5×10^6^ PFU of Adeno-Cre-CMV virus (Viral Vector Core, University of Iowa) by intranasal instillation. *Acidovorax temperans* ATCC 49666 was obtained from the ATCC (Manassas, VA) and cultured in Nutrient Broth (BD Biosciences) at 30 °C with 200 rpm shaking. Two weeks post Ad-cre instillation, mice then were randomly divided to receive six biweekly intranasal instillations of either Sham (1X PBS) or *A. temperans* (1 ×10^9^ CFU) without blinding. Culture inoculum was verified by serial dilution and plating on Nutrient Agar for 48 hours.

### Magnetic resonance imaging

Tumor development was determined 9- and 13-weeks post Ad-cre instillation by magnetic resonance imaging (MRI) with a 3.0 T clinical scanner (Philips Intera Achieva, Best, The Netherlands). Mice were anesthetized then individually imaged using a 40-mm diameter solenoid volume receiver coil (Philips Research, Hamburg, Germany), with anesthesia and air temperature maintained at 1.5–2.0% isoflurane and 34–37 °C, respectively. Multislice T2 weighted turbo spin echo sequence was applied in coronal view with respiratory triggering to minimize motion artifacts. The whole mouse lung was covered by an imaging slab with dimensions 38 × 28 × 16 mm. The images were acquired with a repetition time of 5333 ms, echo time of 45 ms, in plane resolution of 0.188 × 0.188 mm^2^, and a slice thickness 0.5 mm. Lung tumor burden was analyzed by manually segmentation and volume calculation of MRI results using ITK-SNAP 3.8.0 [64, 65].

### Tissue histology

Mice were sacrificed and lungs were harvested at Week 14 post Ad-cre instillation. Lung tissues were bisected longitudinally and fixed in 10% NBF and then embedded in parafilm. Fixed tissue was sectioned at 5 µm thickness for hematoxylin and eosin (H&E) staining. Following staining, whole lung tissues were scanned with Leica AT2 and all proliferative lesions were annotated in a blinded fashion using HALO software v3.4. Tumor area was quantified by calculating total proliferative lesion area by total lung area. Tumor grades were modified from those used by DuPage et al., 2009, with the following criteria. Grade 1 (hyperplasia/small adenoma): proliferation of atypical epithelial cells along with alveoli (adenoma) or projections of epithelial cells along bronchioles (hyperplasia). Grade 2 (large adenoma): enlarged nuclei, prominent nucleoli, distortion of septa, and papillary/solid/mixed pattern. Grade 3 (adenocarcinoma): grade 2 characteristics plus nuclear atypia, cellular polymorphism, and nuclear molding. Grade 4 (invasive adenocarcinoma): grade 3 characteristics plus desmoplasia/invasion and invasive edges bordering blood vessels/pleura.

### Bacterial colony recovery

Bacteria or sham were instilled in WT C57BL/6 mice and harvested at Days 1, 5, or 9 post instillation. Lung tissues were harvested and stored immediately after necropsy in Amies media (ThermoFisher) for bacterial preservation. Lungs were then digested at 37 °C for 40 min using the Mouse Tumor Dissociation kit (Miltenyi Biotec), according to the manufacturer’s instructions. Digested lung tissues were filtered with 40 µM cell strainer to obtain a single-cell suspension. Red blood cells were lysed using RBC lysis buffer (BioLegend), according to the manufacturer’s instruction. The remaining cells were then lysed in 0.05% Triton X-100 for bacterial recovery. Lysate was incubated on ice for 10 min, then serially diluted, and plated in triplicates onto TSB agar containing 5% sheep’s blood. Plates were incubated at 37 °C for 96 h and then colonies were counted. Colony PCR was used to amplify a ~1.4 kb fragment of the 16S rDNA gene using a standard 2X Phusion PC Master Mix (ThermoFisher), according to the manufacturer’s instructions, using the primer pair 16S_27F: 5’-CCTACGGGNGGCWGCAG-3’ and 16S_1491R: 5’-TACGGYTACCTTGTTAYGACTT-3’. Amplicons were Sanger sequenced using the primer 16S_EUB_R2: 5’-CTGCTGCCTCCCGTAGGAGT-3’. Sequencing results were identified using NCBI blastn search.

### Bulk cell RNA sequencing

Mice were sacrificed at Week 10 or 14 post Ad-cre instillation following MRI tumor measurement. Lung tissues were homogenized by bead beating and RNA was extracted using the Quick-DNA/RNA Miniprep kit (Zymo Research), according to the manufacturer’s instructions. RNA quality was determined by Agilent TapeStation and 1 µg of RNA from samples with RIN value ≥ 7.0 were sent for cDNA synthesis and library preparation. Libraries were sequenced on the DNBSeq platform with paired end reads of 2 ×100 bp. Raw reads were cleaned using the BGI pipeline SOAPnuke with cleaned reads aligned to the mm10 genome using Bowtie2 [66]. Differential gene expression was calculated with DESeq2 and gene ontology was conducted using GSEA and IPA [67, 68]. Immune cell infiltration in each mouse was predicted using xCell [21].

### Tissue dissociation and flow cytometry

Four mice per treatment group (sham and *A. temperans*) with MRI measurement were randomly selected without sample size estimation and sacrificed at 14 weeks post Ad-cre instillation and were perfused with PBS + 2 mM EDTA. Lung tissues were processed as above, with single cell suspensions were cryopreserved using 10% DMSO in FBS. Single cell suspensions were later thawed and stained with PE-conjugated anti-CD45 antibody (BD, clone 104, 1:100) and DAPI, then sorted on a BD FACSAria flow sorter and stored on ice.

### Single cell RNA sequencing

Approximately 7000 cells per mouse were targeted for droplet capture by 10X Chromium 3’ Dual Index v3.2 kit. Capture, cDNA synthesis, and library preparation were performed according to the manufacturer’s instructions. Sequencing was performed on Illumina NovaSeq S3 with 10 bp indices i5 and i7, 28 bp R1, and 90 bp R2 length reads. Samples were sequenced to a target depth of 50,000 reads per cell. Basecalling was performed using RTA v2.4.11, demultiplexing with Bcl2fastq v2.20, and read alignment to mouse genome version mm10, tagging, gene and transcript counting, and clustering analysis were performed using CellRanger v6.0.2. The generated filtered matrices were used for downstream analysis.

### Quality control and single cell data analysis

Cells with less than 100 features, more than 2800 features, or greater than 5% mitochondrial reads were excluded using Seurat v4.1.0 [69], followed by removal of fibroblasts (*n* = 590), leaving a total 25,477 cells (14,506 sham and 10,971*A. temperans*) with 20,737 genes detected in at least one cell. Average features per cell was 1397 with an average UMI of 3788 per cell. Passing cell counts were normalized and scaled, then neighbors were clustered using the first 50 dimensions with a resolution of 0.8, resulting in a total of 22 clusters. Cell types were manually assigned using standard marker genes and the ImmGen database [29]. Both the slingshot (v2.2.1) and monocle2 (v2.22.0) packages were used for trajectory and pseudotime analysis [70, 71]. Differentially expressed genes were identified using the Seurat FindMarkers() function and then ssGSEA was performed using the escape v1.4.0 [33]. Cell-cell communication was predicted using CellChat v1.1.2 [72], with the Cell-Cell Interactions and Secreted Signaling datasets selected.

### Immunostaining

For immunohistochemistry, slides were sequentially incubated in fresh xylene, xylene:ethanol (1:1), ethanol, then transferred to cold distilled water. Antigen retrieval (200 mL 1X sodium citrate buffer pH 6) was performed for 20 min at 110 °C then slides were incubated in 1% Triton X-100 prior to washing and peroxidase blocking for 10 min (Vector Laboratories, #SP-2001). Slides were rinsed and blocked for 45 min at RT. After washing, primary antibody (BD, #551459) was incubated overnight at 4 °C. Slides were washed and incubated with a biotinylated secondary antibody for 30 min at RT. Signal was amplified using the VECTASTAIN Elite ABC Kit (Vector Laboratories, #PK-6100) and then developed using ImmPACT DAB solution (Vector Laboratories, #SK-4105). Development was stopped by washing then tissues were counter stained with Mayer’s Hematoxylin, followed by mounting with anti-fade medium.

For immunofluorescence, fresh frozen tissues were sectioned at 5 µm thickness and fixed with cold acetone/methanol (1:1). Tissues were washed prior to blocking with normal goat+donkey serum in 1X PBS for 15 min. Tissues were washed and primary antibodies (CD3: BD #550277, CD4: BioLegend #100402) were incubated overnight at 4 °C. Slides were washed and incubated with conjugated secondary antibodies for 60 min at RT. Tissues were counterstained with DAPI for 2 min, followed by mounting with anti-fade medium.

### TCR-sequencing

Lungs were harvested at 14 weeks post Ad-cre instillation and dissociated. DNA was extracted from lung tissue using the Quick-DNA/RNA Microprep Plus Kit (Zymo Research) and 3 µg was sent for TCRB sequencing using immunoSEQ (Adaptive Biotechnologies).

### Survival analysis

Gene symbols for the murine T17 signature were converted to their human orthologues using biomaRt v2.50.3 [73]. The resulting gene lists were subsetted from expression matrices for GSE30210, GSE31210, GSE50081, and TCGA [74–77]. Human T17 signature scores were tested in a Cox analysis and then cutoff values for low and high expression in each cohort were separately determined using maximally selected rank statistics within survminer v0.4.9 (https://cran.r-project.org/web/packages/survminer/index.html).

### Statistical analysis

Statistical analyses of lung tumor burden and lung weight were performed using GraphPad Prism 8. All sequencing analyses were performed in R v4.1.0 (http://www.r-project.org/). Unless otherwise stated, the t-test was used for comparison between two groups and the ANOVA test was used for comparison between three or more groups. *P* < 0.05 was considered as significantly different.

## Supplementary information

Supplemental Figures

Supplemental Table S1

Supplemental Table S2

Supplemental Table S3

Supplemental Table S4

Supplemental Table S5

Supplemental Table S6

Supplemental Table S7

Supplemental Table S8

## Data Availability

Single-cell and bulk RNA-sequencing data generated in this study were deposited in the NCBI GEO database under the accession numbers GEO: GSE207477 and GSE259408, respectively.

## Code availability

Scripts used in analyses are available upon request from the corresponding author.
