# Supplementary material for: Acidovorax temperans skews neutrophil maturation and polarizes Th17 cells to promote lung adenocarcinoma development
Source: Oncogenesis. 2024 Apr 3;13(1):13. doi: 10.1038/s41389-024-00513-6 (PMC10991269; doi:10.1038/s41389-024-00513-6)
Supplement: Supplementary file 1 — Supplemental Figures [file 41389_2024_513_MOESM1_ESM.pdf]

SUPPLEMENTAL MATERIAL

Supplementary Figure 1

A

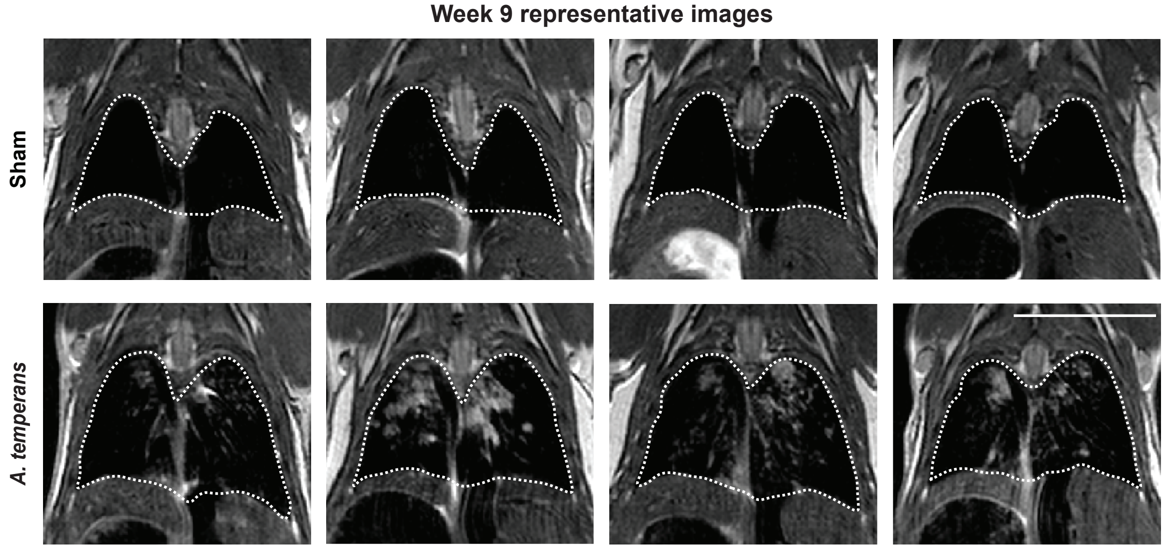

B

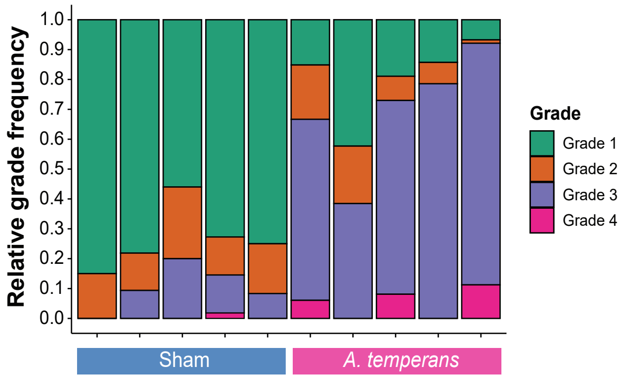

C

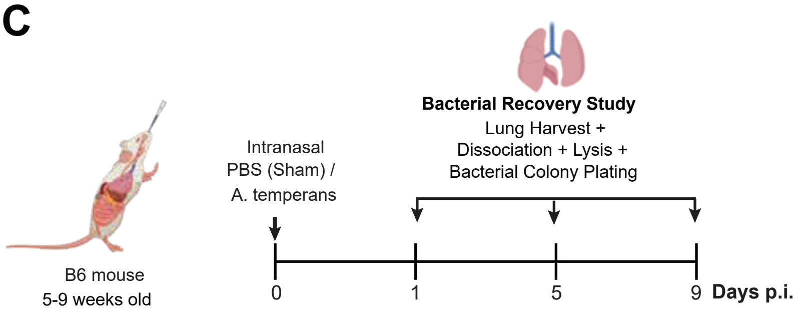

D

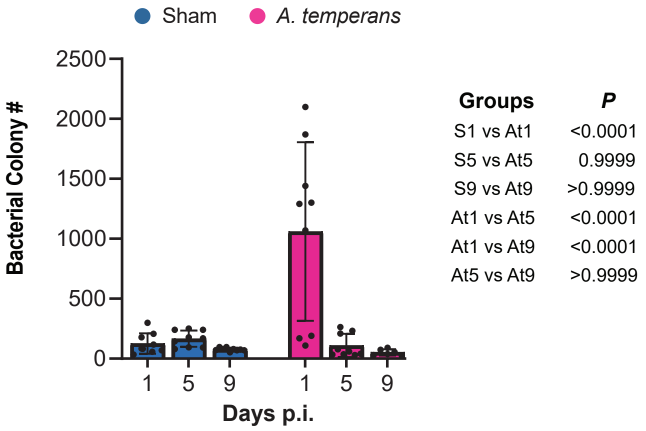

E

	Sham			*A. temperans*			Total
	Day 1	Day 5	Day 9	Day 1	Day 5	Day 9	
Acidovorax	0	0	0	6	0	0	6
Escherichia	0	2	0	0	0	0	2
Lactobacillus	3	0	2	1	3	2	11
Lactococcus	2	0	0	0	0	0	2
Rodentibacter	0	1	0	3	3	2	9
Staphylococcus	0	0	0	2	0	0	2
Streptococcus	4	5	3	1	0	2	15
Total	9	8	5	13	6	6	47

Fig. S1 – *Acidovorax temperans* accelerates early tumor development but does not persist in lungs.

A MRI images of sham (1X PBS) (top) and *A. temperans* (bottom) instilled mice at 9 weeks post Adcre instillation. **B** Relative tumor grade abundance per mouse from H&E staining. **C** Experimental outline for bacterial recovery study. **D** Colony numbers after recovery on TSB agar at Days 1, 5, and 9 post *A. temperans* instillation. **E** Single colonies from **(D)** were picked and inoculated in NB broth prior to Sanger sequencing of the 16s rRNA gene. Genera were assigned by closest blastn result.

Supplementary Figure 2

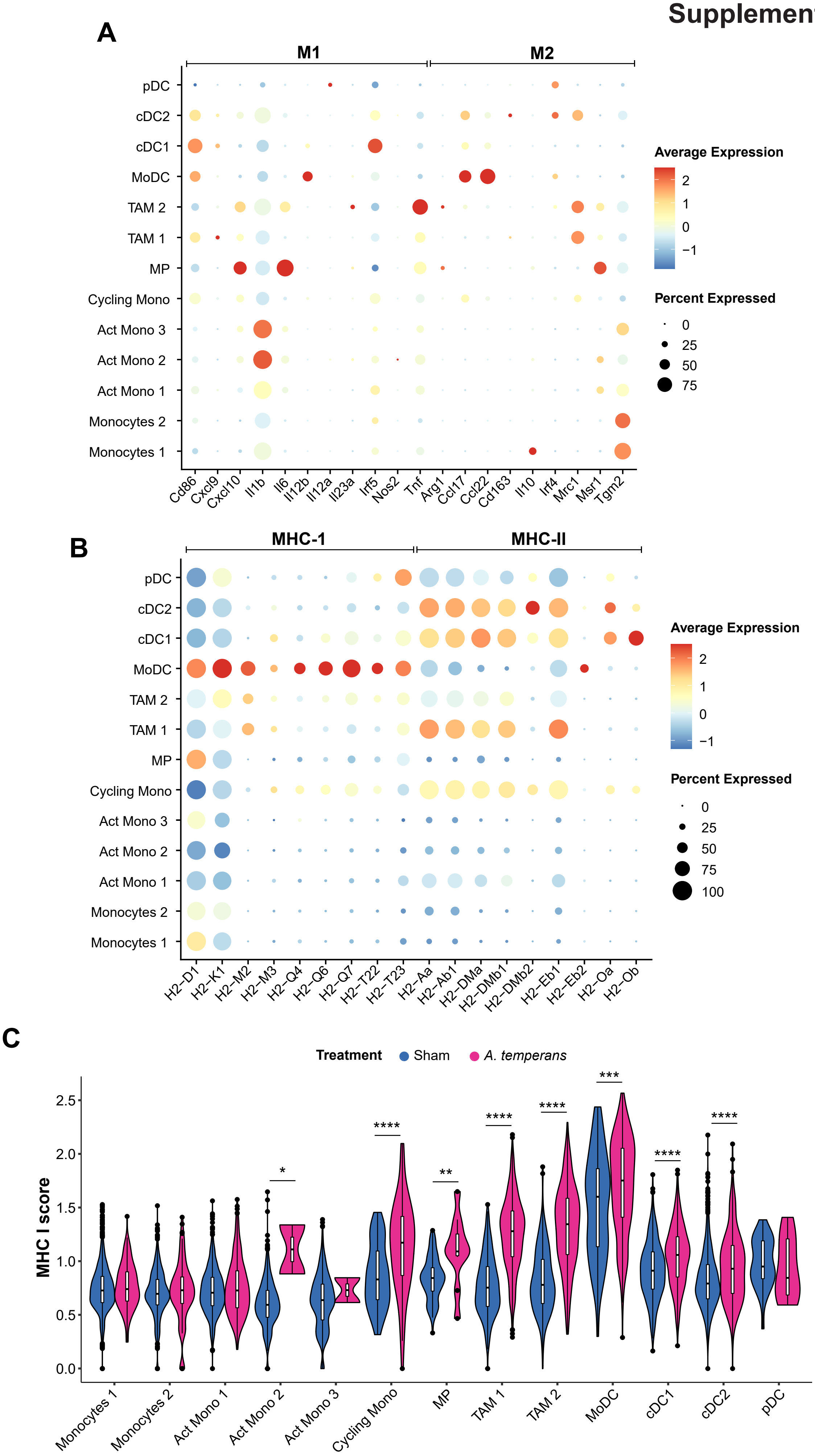

Fig. S2 – MHC I is broadly expressed in MoMaDCs and upregulated in TAMs.
A Dotplot of M1 and M2 macrophage marker gene expression in MoMaDCs. **B** Dotplot of individual MHC class I and II gene expression in MoMaDCs. **C** Comparison of average expression of each MHC I component gene (*H2-D1, -K1, -M2, -M3, -Q4, -Q6, -Q7, -T22, -T23*) by treatment for each cell type. Data presented as median value plus quartiles for boxplots, n.s. not significant, * $p < 0.05$, ** $p < 0.01$, *** $p < 0.001$, **** $p < 0.0001$.

Supplementary Figure 3

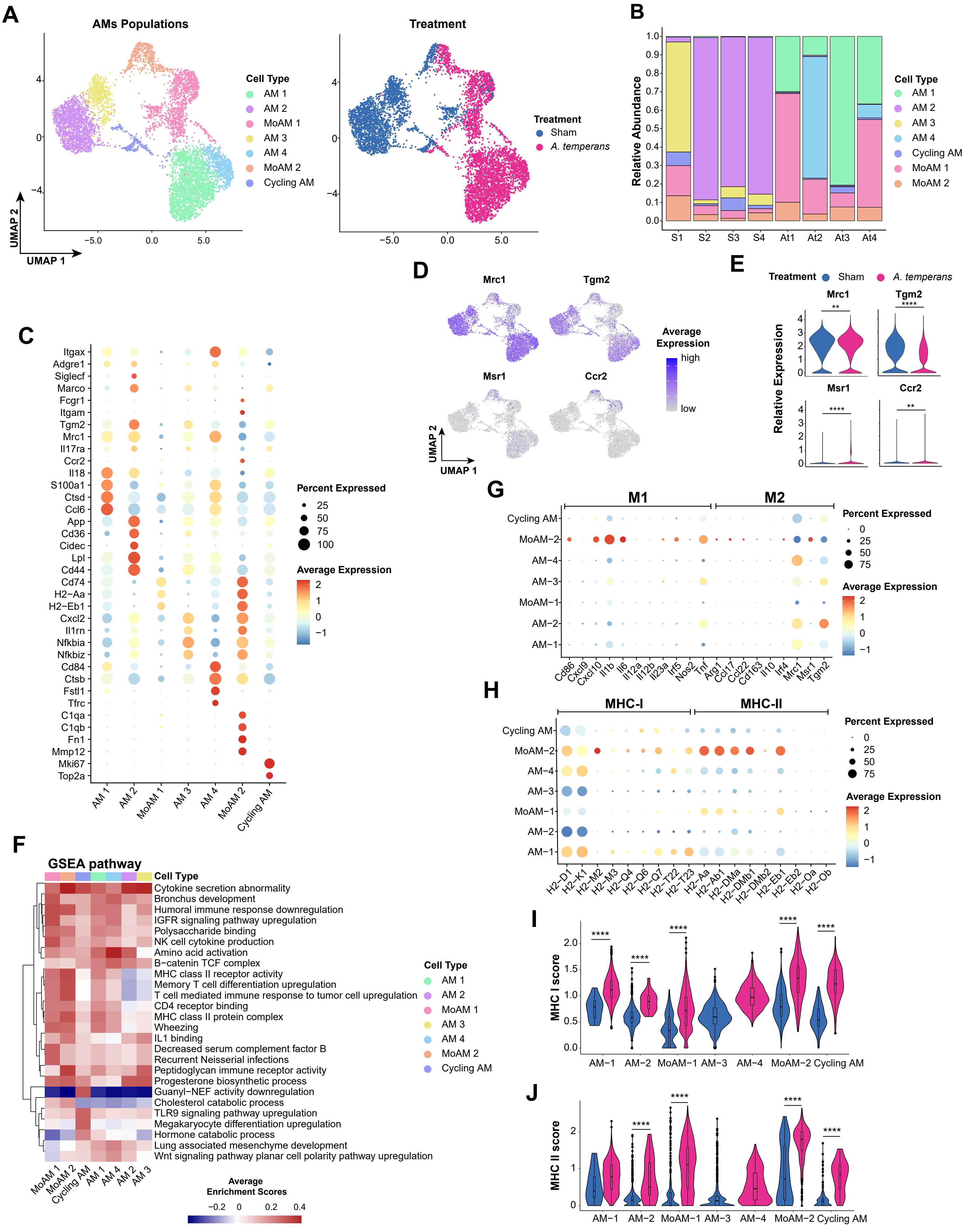

Fig. S3 – *Acidovorax temperans* induces MHC II upregulation in alveolar macrophages. **A** UMAP plots of alveolar macrophages (AMs) cell types (left) and treatment groups (right). **B** Barplot of the relative abundance for each cell subtype by individual mouse with sham (S, n = 4) or *A. temperans* (At, n = 4) instillation. **C** Dotplot of marker genes for each cluster. **D** Density plot for AM marker genes (*Mrc1* and *Tgm2*) and monocyte-derived AMs (*Msr1* and *Ccr2*). **E** Quantification of the total expression of marker genes by treatment. **F** ssGSEA heatmap of average normalized enrichment scores for each AM cluster. **G** Dotplot of M1 and M2 macrophage marker gene expression in AMs. **H** Dotplot of individual MHC class I and II gene expression in AMs. **I** Comparison of average expression of each MHC I component gene (*H2-D1, -K1, -M2, -M3, -Q4, -Q6, -Q7, -T22, -T23*) by treatment for each cluster. **J** Comparison of average expression of each MHC II component gene (*H2-Aa, -Ab1, -DMa, -DMb1, -DMb2, -Eb1, -Eb2, -Oa, -Ob*) by treatment for each cell type. Data presented as median value plus quartiles for boxplots, ** $p < 0.01$, **** $p < 0.0001$.

Supplementary Figure 4

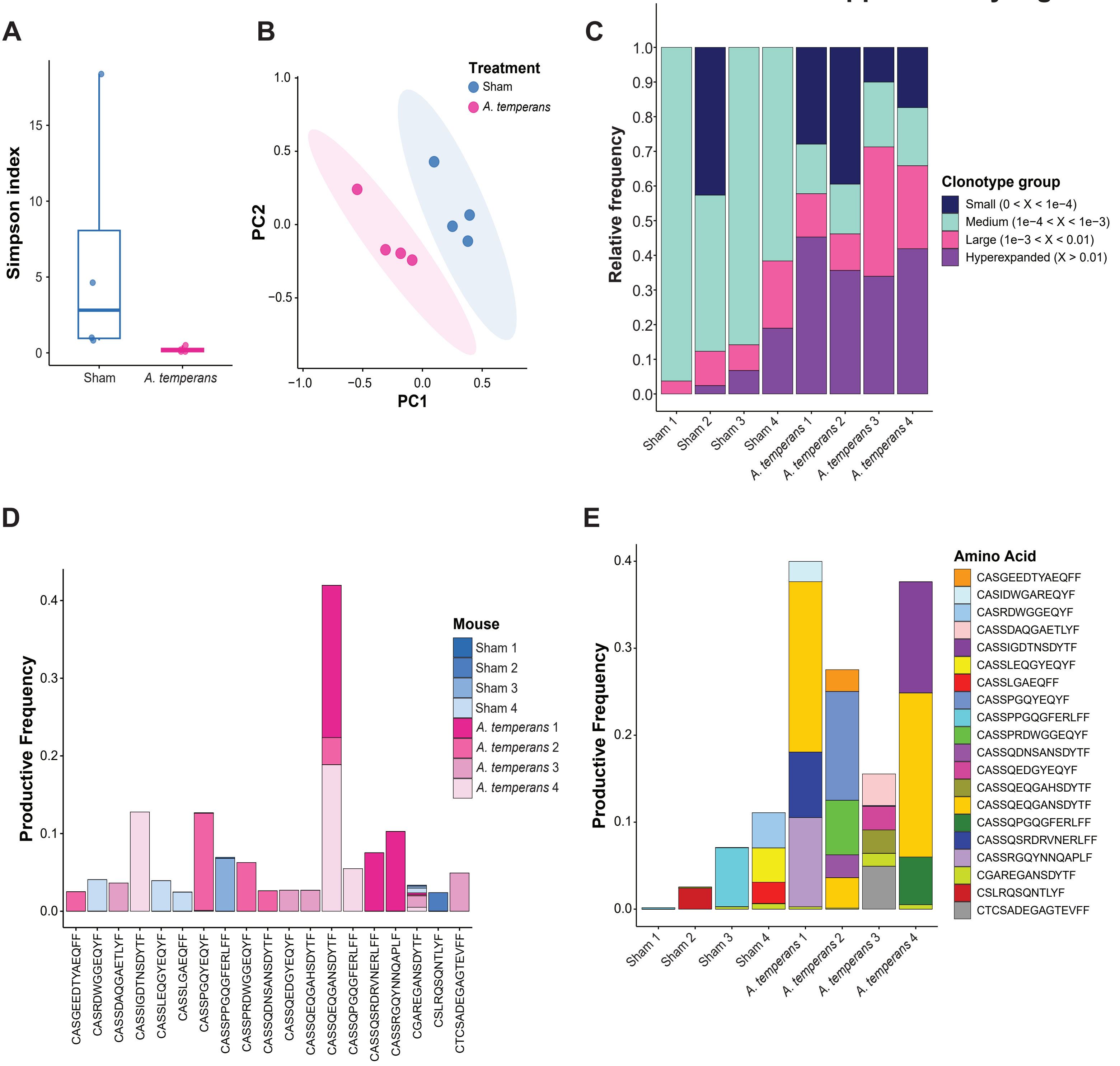

Fig. S4 – TCR clonality is decreased in response to *A. temperans*.
A Alpha diversity (Simpson index) of TCR clonotypes in sham (n = 4) and *A. temperans* (n = 4) instilled mice. **B** Beta diversity (Bray-Curtiss) of TCR clonotypes in sham and *A. temperans* instilled mice. **C** Barplot of relative frequency of TCR clonotypes by expansion. **D** Relative productive frequency of the top 20 amino acid TCR recombinations per mouse. **E** Relative productive frequency of each mouse per top 20 amino acid TCR recombinations.

Supplementary Figure 5

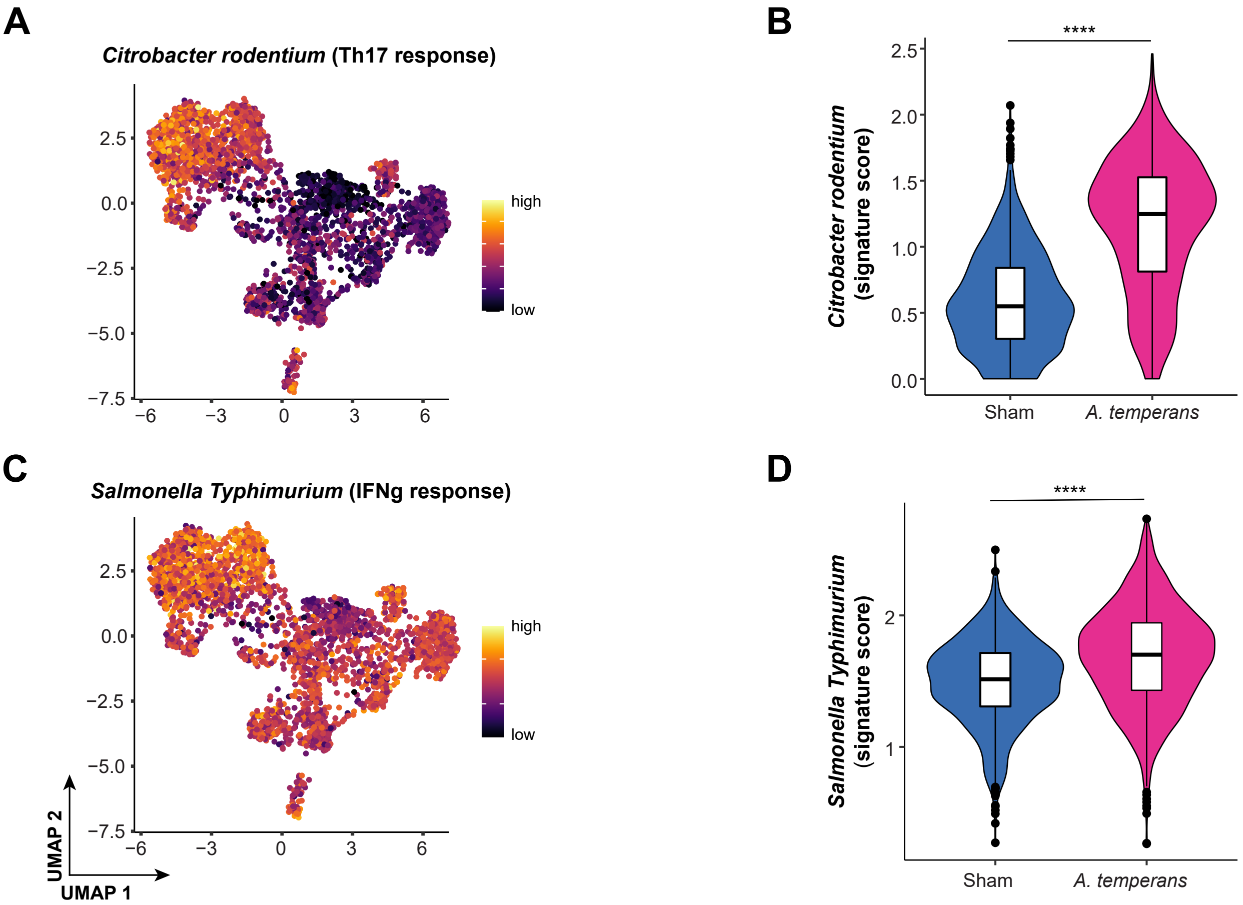

Fig. S5 – Bacterial infection datasets reveal *A. temperans* induces specific T_H17 and general IFN-γ response in T cells.
A UMAP plot of a T_H17 response gene signature in a *Citrobacter rodentium* infection model (44) within T cell subtypes. **B** Violin plot of the expression of the *C. rodentium* gene signature by treatment group. **C** UMAP plot of an IFN-γ response gene signature in a *Salmonella enteria* serovar Typhimurium infection model (44) within T cell subtypes. **D** Violin plot of the expression of the *Salmonella* Typhimurium gene signature by treatment group. Data presented as median value plus quartiles for boxplots, **** $p < 0.0001$.

Supplementary Figure 6

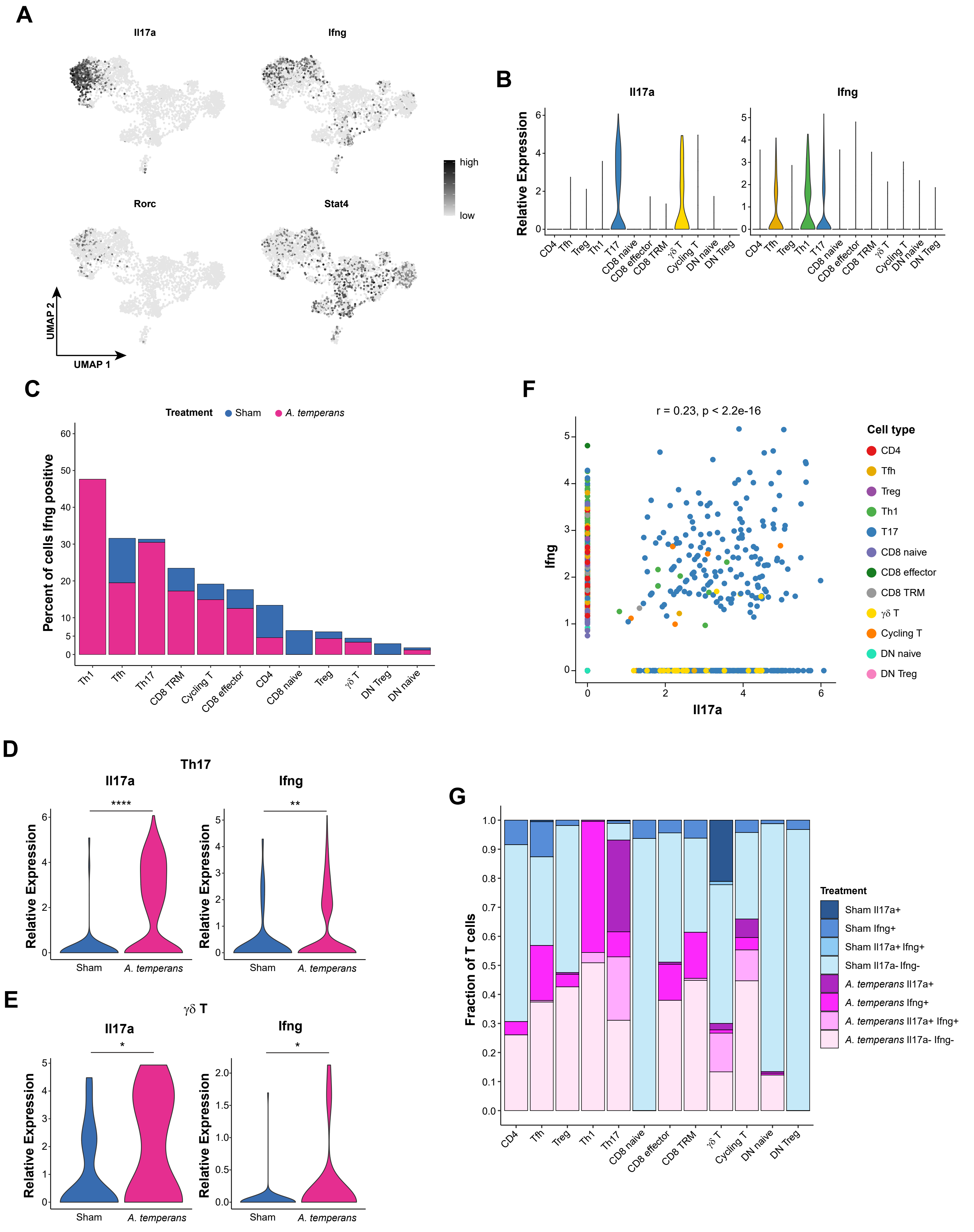

Fig. S6 – Both IFN-γ and IL-17 are expressed in response to *A. temperans*.
A Density plot of the cytokine *Il17a* and its transcription factor *Rorc* (left) and the cytokine *Ifng* and its transcription factor *Stat4* (right) within T cells. **B** Expression level of *Il17a* and *Ifng* by T cell subtype. **C** Percent of cells *Ifng* positive per T cell subtype and treatment group. **D, E** Expression level of *Il17a* (right) and *Ifng* (left) within the **(D)** Th17 and **(E)** γδ T clusters by treatment group. **F** Correlation of *Il17a* and *Ifng* in all T cells. **G** Barplot of *Il17a/Ifng* expression per mouse. Data presented as median value plus quartiles for boxplots, * $p < 0.05$, ** $p < 0.01$, **** $p < 0.0001$.

Supplementary Figure 7

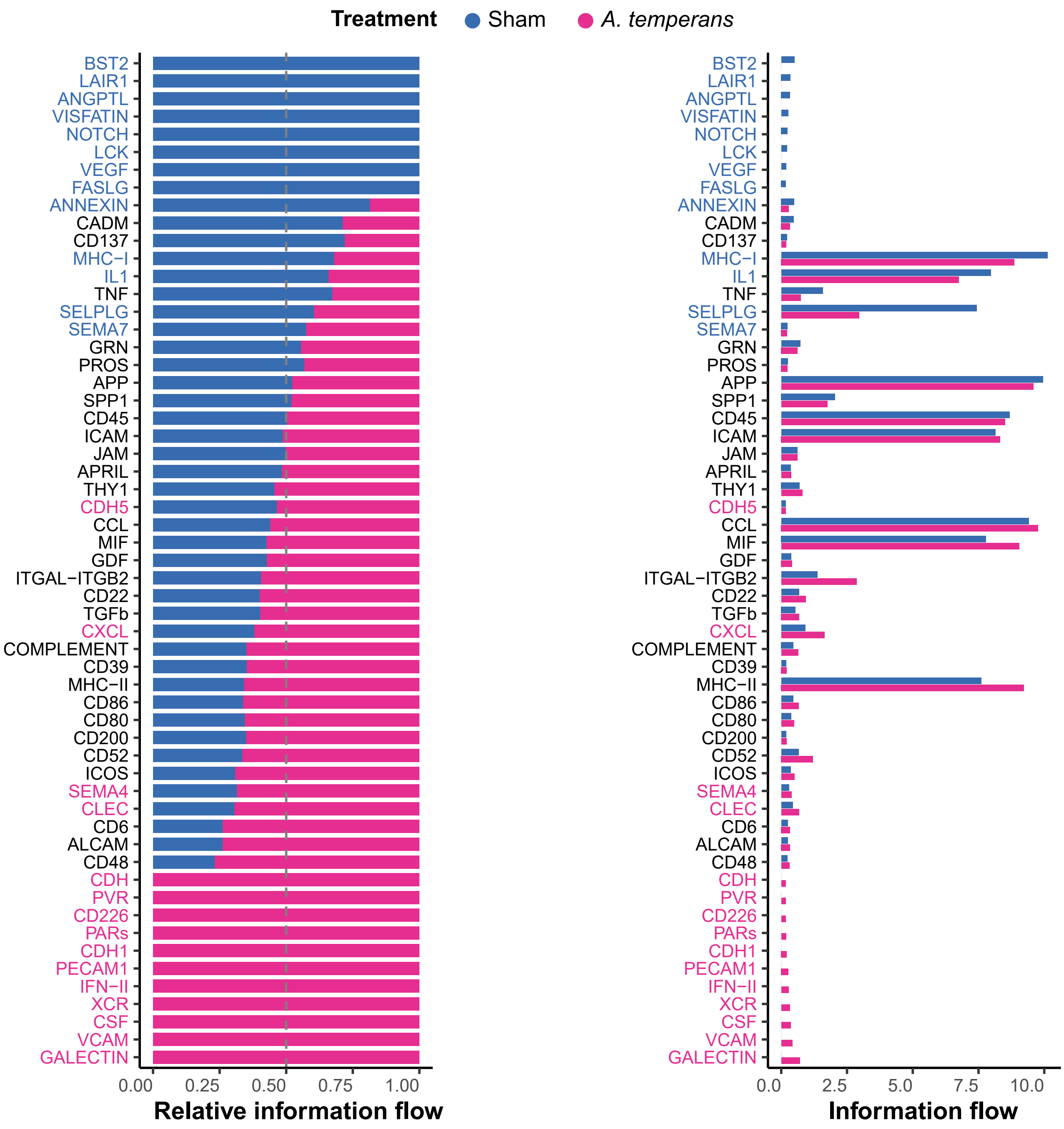

Fig. S7 – Specificity of ligand-receptor signaling pathways by treatment group.
Relative (left) and absolute (right) contribution of aggregate signaling pathways, ordered from sham-enriched (top, blue) to *A. temperans*-enriched (bottom, pink).

Supplementary Figure S8

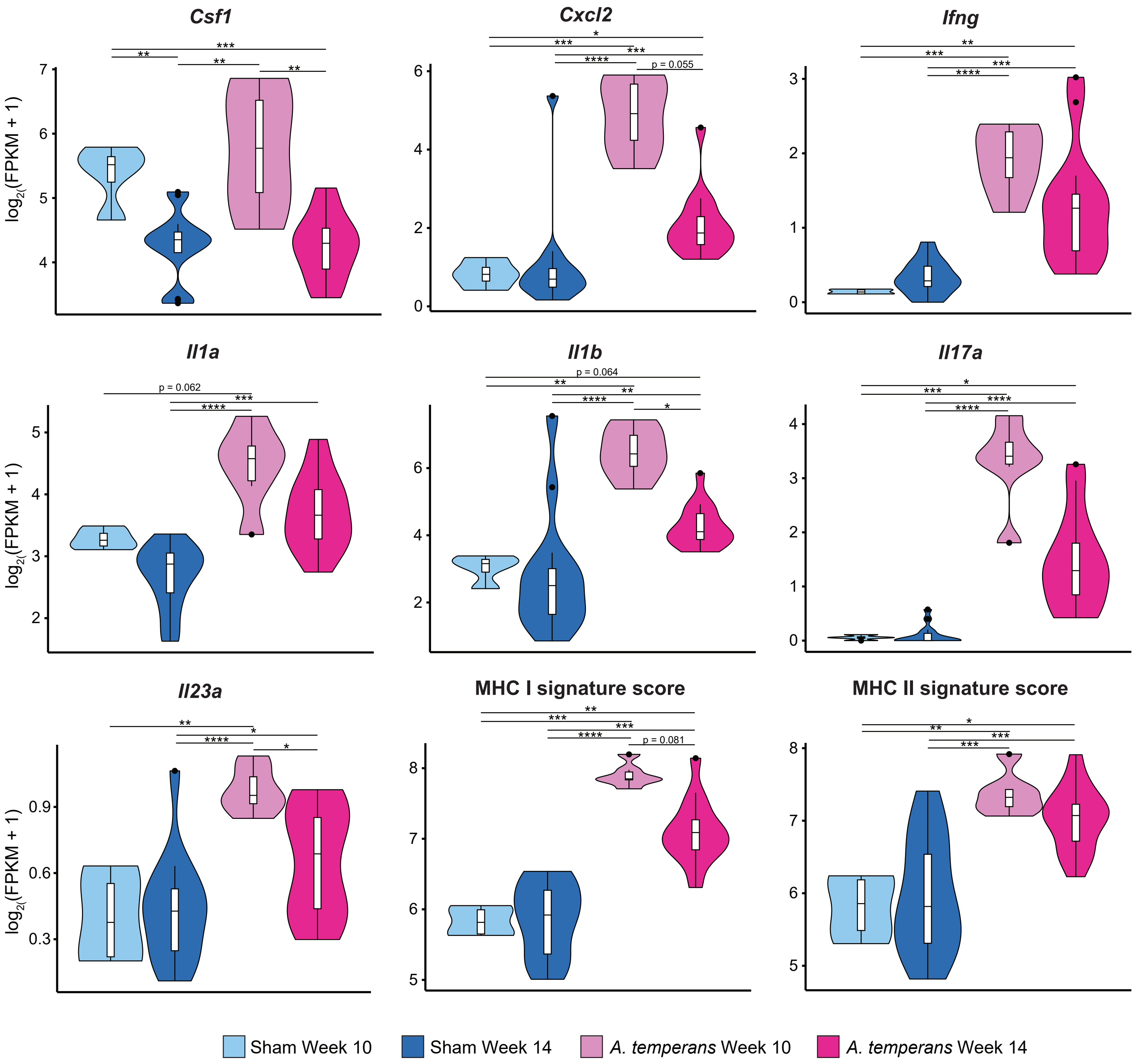

Fig. S8 – Bulk RNA-seq results scRNA-seq and show higher levels at earlier timepoint in *A. temperans* instilled mice.
Bulk RNA-seq validation of cytokines identified by scRNA-seq, with sham week 10 (n = 4), sham week 14 (n = 14), *A. temperans* week 10 (n = 15), and *A. temperans* week 14 (n = 6) instilled mice. Expression values graphed log_2-transformed FPKM values. MHC I and II signature scores presented as average expression of individual MHC genes found in scRNA-seq dataset. MHC I: *H2-D1, H2-K1, H2-M2, H2-M3, H2-Q4, H2-Q6, H2-Q7, H2-T22, H2-T23*. MHC II: *H2-Aa, H2-Ab1, H2-DMa, H2-DMb1, H2-DMb2, H2-Eb1, H2-Eb2, H2-Oa, H2-Ob*. Boxplots indicate median and quartile scores. * $p < 0.05$, ** $p < 0.01$, *** $p < 0.001$, **** $p < 0.0001$.

Table S1 – Main cell type marker genes.

Table S2 – MoMaDC marker genes.

Table S3 – Alveolar macrophage marker genes.

Table S4 – Neutrophil marker genes.

Table S5 – T cell marker genes.

Table S6 – Common Th17 and γδ T marker genes used for pan T17 signature.

Table S7 – Cell-cell interactions increased in *A. temperans* mice.

Table S8 – Cell-cell interactions decreased in *A. temperans* mice.
